# Airway management of a child with multiple congenital anomalies

**DOI:** 10.4103/0019-5049.63642

**Published:** 2010

**Authors:** Rajeev Sharma

**Affiliations:** Department of Anesthesiology, ESI Hospital, Rohini, New Delhi, India

Sir,

I read with interest the recent case report on anaesthetic management of a child with multiple congenital anomalies scheduled for cataract extraction by Kulkarni *et al*.[1] There are several fundamental concerns regarding the anaesthetic management of the presented case.

Firstly, there was a history of recurrent respiratory distress, noisy breathing and hacking cough. The infant had a weak cry also. Fundamentally, when one encounters a child with weak cry, recurrent respiratory distress and hacking cough, one must rule out associated airway anomalies.[2] Laryngeal anomalies can present as drooling of saliva too as was present in this case.[3]

This was even more essential in view of the associated mental retardation and choroid-plexus cyst, as there is a strong association of CNS abnormalities with vocal cord palsy, moreso in association with cardiac anomalies.[3]

The use of nitrous oxide during induction for both the surgeries is questionable because the child was already in respiratory distress before the first surgery and airway stenosis was documented before the second surgery especially given the fact that no detailed airway evaluation was performed.

The authors administered succinylcholine after initial failed intubation attempts. One has to be very cautious while using muscle relaxants in such infants after failed intubation attempts because a partial airway obstruction caused by the airway anomaly could rapidly progress to total airway obstruction due to edema caused by airway manipulations. This could rapidly make the mask ventilation impossible. This was partly reflected in the postoperative period when the child developed stridor.

Fourth, tracheal intubation was not performed postoperatively even when there was stridor, respiratory distress and oxygen desaturation. The edema could have progressed to total airway occlusion. We must err on side of safety especially when even a size 2.0 tube could be introduced only with resistance after multiple failed attempts.

Fifth, postcricoid narrowing on radiology is not confirmatory of isolated subglottic stenosis. Laryngeal webs frequently have a subglottic extension[4] that may present as subglottic stenosis radiologically. Therefore, the subglottic stenosis must have been evaluated by referring to some center where bronchoscopes are available. Rigid bronchoscopy performed under general anaesthesia gives a better view of airway than flexible endoscopy, especially for the part below the level of vocal cords[2] (site, thickness and the extent of web). When one airway anomaly is found, it is critical to rule out others since subglottic stenosis results due to failure of recanalization of embryonic mesoderm which can have varied presentations including laryngeal web, laryngomalacia, etc.[3]

Finally, the literature is abound with iatrogenic, post-intubation laryngeal webs and stenosis. Therefore, proceeding with intubation without proper airway evaluation seems against the basics of safe airway management.
